# Regular Muscle Electrical Stimulation Could Act Favorably On Bone Mineral Density in Healthy Aged Subjects

**DOI:** 10.3389/fphys.2018.01035

**Published:** 2018-07-31

**Authors:** Thierry Paillard

**Affiliations:** University of Pau and Pays de l'Adour, Pau, Pyrénées-Atlantiques, France

**Keywords:** muscle electrical stimulation, bone mineral density, osteoporosis, osteopenia, aging, elderly, old subject, bone mass

Bone is a living tissue undergoing perpetual remodeling. Bone cells are daily created while other cells disappear. Two antagonistic pathways interact with each other (Wnt pathway or wingless-related integration site and RANK/RANKL/OPG pathway or receptor activator of nuclear factor-kappa B/receptor activator of nuclear factor-kappa B ligand/osteoprotegerin) and ensure balance between bone formation and bone resorption in young adults (Mitchell and Streeten, 2013). These two pathways regulate the activity of osteoblasts (responsible for the formation of bone tissue) and osteoclasts (responsible for the resorption of bone tissue).

With advancing age, the activity of osteoclasts progressively overtakes that of osteoblasts so that the bone mineral density (BMD) continually decreases, which is inevitable even in healthy subjects (Paillard, 2014). As the number and activity of osteoblasts decreases, some individual trabeculae disappear or undergo perforations (Marie and Kassem, 2011). This causes, on the one hand, a reduction of the space necessary for the formation of new bone cells, and on the other hand, a disorganization of the trabecular network. The cortical bone also undergoes an alteration which is mainly provoked by increased porosity from both an increase in resorption cavities and an accumulation of incompletely closed osteons (Chen et al., 2013). Thus, aging is characterized by a reduction in mass and a change in the architecture of bone tissue. These histological changes in bone are defined by the term osteopenia and are also related to a slowing of the speed of the bone remodeling process. If the reduction of the value of BMD is greater than 2.5 standard deviations in comparison with normal values in young adults, the term of osteopenia is substituted by that of osteoporosis (Paillard, 2014). In this context, the mechanical resistance of bone is jeopardized. Since the risk of falling due to the alteration of the postural function also increases with age, the risk of fracture increases logically with age (Nitz et al., 2013).

In order to boost the osteoblast activity and to reduce the osteopenia in healthy aged subjects, bone should undergo mechanical deformations from any kind of external force (e.g., shock, pressure, mechanical stress). To this end, regular physical exercise is a physiological method (with appropriate diet) that can mitigate the effects of normal (non-pathological) bone demineralization, especially in healthy older subjects (Alghadir et al., 2016; Watson et al., 2018). Indeed, physical exercise induces mechanical constraints (i.e., mechanical stress) generating bone deformation which stimulates osteogenesis and favors bone remodeling (Srinivasan et al., 2012).

However, very old people (even healthy ones) are often either unable or unwilling to perform conventional exercise programs (Paillard, 2018). Evidence suggests that the lower the physical activity volume, the lower the bone mineral density. Hence, a vicious circle is set up and the osteopenia process is amplified because of the lack of physical activity and mechanical constraints applied on bone. In this context, only an artificial technique not requiring any effort to generate (passive) repetitive muscle contractions likely to exert mechanical stimuli on bone could break this vicious circle. Regular neuromuscular electrical stimulation (NMES) may be a feasible alternative intervention to enhance BMD. Nevertheless, physical exercise achieved in condition of body discharge i.e., the body is not in contact with the ground (e.g., swimming, cycling) or in a static condition i.e., without any dynamic and/or ample movement (e.g., stretching, balance) does not stimulate (or very weakly) osteogenesis (Paillard, 2014). This author specifies that the osteogenic function of aerobic training (e.g., walking, running) is effective only if the intensity of exercise is high (i.e., generating strong impacts on the ground) and that of strength training is effective only if the completed muscular contractions are dynamic and carried out with heavy loads. On the basis of findings mentioned above, NMES is unlikely to have an ostegenic function since it is practiced in a static condition (often seated on a chair i.e., in condition body discharge) and does not engender any evident mechanical stress on bone.

However, although the osteogenic effects of NMES remain uncertain, the question still deserves to be raised since the regular/chronic application of NMES has numerous advantages related to the muscle function and thus in the fight against sarcopenia (Paillard et al., 2004; Kern et al., 2014; Barberi et al., 2015; Paillard, 2018). On the one hand, NMES can be clinically and preventively substituted for voluntary exercise in older subjects to stimulate their muscle function (Von Stengel et al., 2015). On the other hand, therapists consider that the anti-osteoporosis and anti-sarcopenia roles of exercise are often inseparable in older subjects (Paillard et al., 2005; Bettis et al., 2018). Hence, it seems appropriated to analyze the effects of regular NMES sessions (NMES training) on BMD (especially as regards the lower limb) in healthy older subjects to provide current information accompanied with an argued opinion.

It is important to note that the effects of NMES on BMD were largely studied in animals as well as in human in different physiopathological contexts such as musculoskeletal injuries, spinal cord injury, fractured bone (healing bone), and in situations of microgravity (e.g., Hamanishi et al., 1995; Park and Silva, 2004; Peng et al., 2005; Shields and Dudley-Javoroski, 2006; Groah et al., 2010). The effects on BMD were also analyzed as part of the utilization of the functional electrical stimulation which helps the completion of voluntary movements in subjects presenting motor deficiency (Leeds et al., 1990; Dolbow et al., 2012; Chang et al., 2013). In return, the effects of NMES on the BMD in healthy older subjects were very rarely tackled.

In fact, the few studies that have dealt with this topic showed that even if the NMES does not engender as much mechanical constraints on bone as the dynamic and intense physical activity, it could be nevertheless beneficial to osteogenesis. In 38 sedentary osteopenic 70 year-old women who benefited from 3 sessions every 14 days for 1 year of whole-body NMES, Von Stengel et al. (2015) reported a borderline significant effect (*p* = 0.051) for the lumbar spine BMD but not for the femoral neck site (absorptiometry measures) in comparison with 38 control subjects. After 4 sessions a week of NMES on quadriceps femoris for 6 weeks in 62 to 75 year-old women, Paillard et al. (2003) however observed no enhancement of BMD at the level of different body regions (e.g., total body, femoral, lumbar, legs). By contrast, in this study, when the NMES was superimposed onto voluntary muscular contractions (climbing and coming down 300 stairs per session) the BMD enhanced more on the trochanters and the whole legs than NMES or voluntary muscular contractions carried out alone. The superimposed activity (stair-climbing and NMES practiced simultaneously) would induce a more important and/or constant traction on femoral bone than each activity practiced alone which could explain why it stimulated the osteogenesis more. In fact, when increases in the BMD are observed, they are always noticed on the sites having undergone strong mechanical loads (Gutin and Kasper, 1992). With functional electrical stimulation, Bélanger et al. (2000) have already showed that the BMD increased on the specific site where loading was mainly applied.

Regarding the NMES, the duration of the training programs should be relatively long to increase the BMD as was previously reported with voluntary physical activity (Layne and Nelson, 1999; Paillard, 2014). Dolbow et al. (2013) infered that the NMES may help slow the process of bone loss and increase the bone mass density after long applications (minimum of 12 months) in aged spinal cord injury subjects. A long duration would be specifically recommended for older women since it is particularly difficult to increase BMD in this population through physical activity (Blumenthal et al., 1991; Paillard, 2014). However, a shorter training period of NMES could increase the BMD and/or limit its reduction related to advancing age when it is superimposed onto voluntary muscular contractions (Paillard et al., 2003). The effects of NMES superimposed onto vonluntary muscular contraction would indeed deserve to be tested on a longer period (>6 weeks of training) to confirm or not its relevance on BMD of lower limbs. Moreover, Von Stengel et al. (2015) specified that in order to favor BMD through NMES training, it would be advisable to employ a high frequency and high intensity current. From this viewpoint, the optimal parameters of the current required to enhance muscle strength seem to be suitable for improving BMD (for instance, frequency >50 Hz, intensity matching the maximum tolerance threshold of subjects). Indeed, one can theoretically suggest that the stronger the muscle contraction, the greater the mechanical constraint on the bone where the muscle is inserted. It is known that the greater the bone mechanical constraint (i.e., heavier load), the higher the BMD after training periods (Paillard, 2014).

The effects of NMES on the osteogenesis or the reduction of bone loss related to advancing age might be explained by the induction of mechanical and humoral factors linked to electro-induced contractions (Figure 1). It was shown with animal models and human subjects that mechanical stimuli of osteoblasts induces the secretion of growth factors including insulin-like growth factor (IGF), vascular endothelial growth factor (VGEF), transforming growth factor (TGF)-β, and the bone morphogenetic protein (BMP) that are considered to be the principal local regulators of osteogenesis (Papachroni et al., 2009; Tamaki et al., 2014). Feng et al. (2016) specified the NMES effectively downregulated myostatin mRNA, upregulated mechano growth factor (MGF) and IGF-1 mRNA expression which mitigated cortical bone loss. Physiologically, the acute application of NMES contributes to increasing local blood flow and augmenting circulation of the components necessary for bone formation. NMES sessions regularly repeated could favor bone formation.

**Figure 1:**
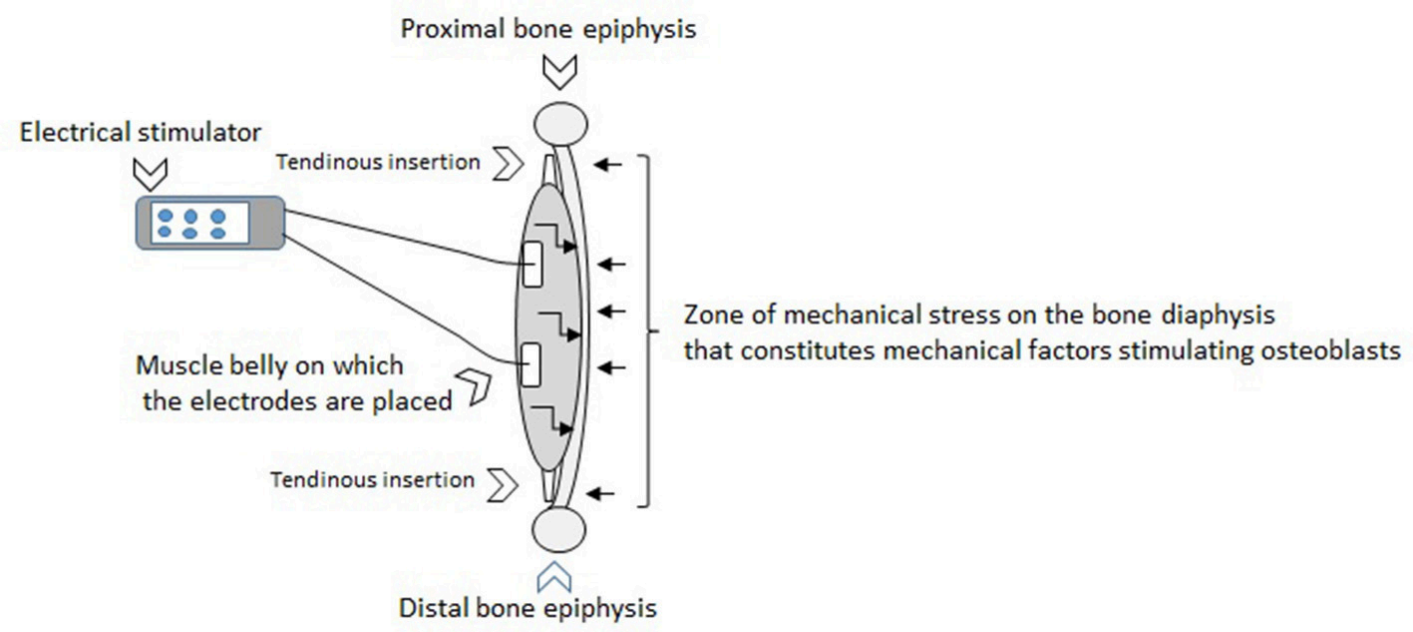
The effects of NMES on the osteogenesis or the reduction of bone loss related to advancing age might be explained by the induction of mechanical and humoral factors linked to electro-induced contractions. The figure above illustrates the application of electrical stimulation through surface electrodes that generates muscle contractions. This muscle action can be characterized by tractions of tendons on the bone extremities. These tractions engender a mechanical stress along the bone diaphysis on the opposite side to the location of the muscle in relation to the axis of the bone (represented by small right arrows on the figure toward the bone diaphysis). The zone of mechanical stress on the bone diaphysis matches mechanical factors stimulating osteoblasts. It is known that the higher the intensity of the current, the stronger the muscle contraction. It turns out that the stronger the muscle contraction, the greater the mechanical stress on the bone where the muscle is inserted. The ostegenesis would be directly proportional to the value of the mechanical stress induced. Therefore, the osteogenesis could be at least partially related to the intensity of the current. This figure also illustrates the fact that the NMES induces acute physiological adaptations including secretions of local growth factors (represented by small broken arrows on the figure into the muscle toward the bone diaphysis). These whole stimuli regularly generated through the chronic application of NMES would be currently considered as positive on the BMD.

Initially, the effects of NMES on BMD in healthy older subjects seemed unlikely but the available results in the literature appear to be positive even if they are not yet formally established and many of the mechanisms are not yet explained. This topic deserves to be explored further as part of the prevention of osteopenia and osteoporosis in healthy older subjects.

## Author contributions

The author confirms being the sole contributor of this work and approved it for publication.

## Conflict of interest statement

The author declares that the research was conducted in the absence of any commercial or financial relationships that could be construed as a potential conflict of interest.
